# 1-(4-Fluoro­phen­yl)-2-(1*H*-imidazol-1-yl)ethanol

**DOI:** 10.1107/S1600536811053505

**Published:** 2011-12-17

**Authors:** Dong-liang Liu, Chen Li, Xin Tian, Song Li, Tao Xiao

**Affiliations:** a Department of Applied Chemistry, College of Science, Nanjing University of Technology, Nanjing 210009, People’s Republic of China

## Abstract

In the title compound, C_11_H_11_FN_2_O, the dihedral angle between the mean planes of the two rings is 1.30 (4)°. In the crystal, O—H⋯N hydrogen bonds link the mol­ecules into chains along the *b* axis.

## Related literature

For related compounds containing a 2-(1*H*-imidazol-1-yl)-1-phenyl­ethanol fragment, see: Porretta *et al.* (1993 ▶). For related structures, see: Tao *et al.* (2007 ▶); Liu *et al.* (2011 ▶). For standard bond lengths, see: Allen *et al.* (1987 ▶).
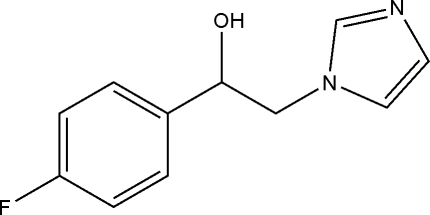


## Experimental

### 

#### Crystal data

C_11_H_11_FN_2_O
*M* *_r_* = 206.22
Monoclinic, 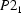

*a* = 7.1220 (14) Å
*b* = 5.4690 (11) Å
*c* = 12.981 (3) Å
β = 98.13 (3)°
*V* = 500.53 (19) Å^3^
*Z* = 2
Mo *K*α radiation
μ = 0.10 mm^−1^
*T* = 293 K
0.30 × 0.20 × 0.10 mm

#### Data collection

Enraf–Nonius CAD-4 diffractometer
Absorption correction: multi-scan (North *et al.*, 1968 ▶) *T*_min_ = 0.970, *T*_max_ = 0.990
1992 measured reflections
1024 independent reflections
876 reflections with *I* > 2σ(*I*)
*R*_int_ = 0.029
3 standard reflections every 200 reflections  intensity decay: 1%

#### Refinement

*R*[*F* ^2^ > 2σ(*F* ^2^)] = 0.041
*wR*(*F* ^2^) = 0.124
*S* = 1.00
1024 reflections
139 parameters
1 restraint
H atoms treated by a mixture of independent and constrained refinement
Δρ_max_ = 0.13 e Å^−3^
Δρ_min_ = −0.18 e Å^−3^

### 

Data collection: *CAD-4 EXPRESS* (Enraf–Nonius, 1994 ▶); cell refinement: *CAD-4 EXPRESS*; data reduction: *XCAD4* (Harms & Wocadlo, 1995 ▶); program(s) used to solve structure: *SHELXS97* (Sheldrick, 2008 ▶); program(s) used to refine structure: *SHELXL97* (Sheldrick, 2008 ▶); molecular graphics: *PLATON* (Spek, 2009 ▶); software used to prepare material for publication: *SHELXTL* (Sheldrick, 2008 ▶).

## Supplementary Material

Crystal structure: contains datablock(s) I, global. DOI: 10.1107/S1600536811053505/zq2137sup1.cif

Structure factors: contains datablock(s) I. DOI: 10.1107/S1600536811053505/zq2137Isup2.hkl

Supplementary material file. DOI: 10.1107/S1600536811053505/zq2137Isup3.cml

Additional supplementary materials:  crystallographic information; 3D view; checkCIF report

## Figures and Tables

**Table 1:** Hydrogen-bond geometry (Å, °)

*D*—H⋯*A*	*D*—H	H⋯*A*	*D*⋯*A*	*D*—H⋯*A*
O—H0*A*⋯N2^i^	0.87 (4)	1.90 (4)	2.762 (4)	171 (5)

Symmetry code: (i) 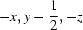.

**Fig. 1.**
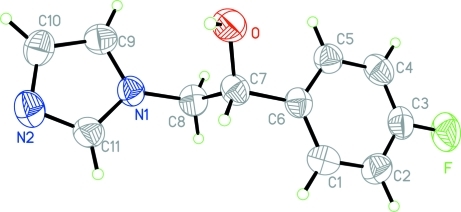
The molecular structure of the title compound with displacement ellipsoids drawn at the 50% probability level.

**Fig. 2.**
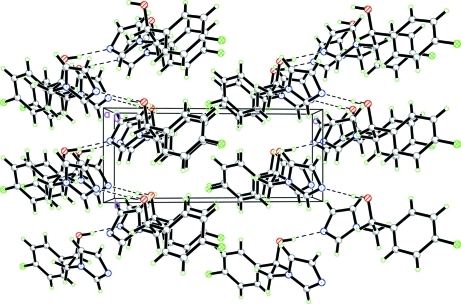
A packing diagram of the title compound viewed down the b axis. Hydrogen bonds are shown as dashed lines.

## supplementary crystallographic information

### Comment

The title compound, C_11_H_11_ON_2_F, is the key intermediate in the synthesis of a new kind of antifungal drug (Porretta *et al.*, 1993). As a part of our ongoing studies (Tao *et al.*, 2007; Liu *et al.*, 2011), the crystal structure determination of the title compound has been carried out in order to elucidate its molecular conformation. The molecular structure of the title compound is shown in Fig. 1. Bond lengths (Allen *et al.*, 1987) and angles are within normal ranges. The dihedral angle between the mean planes of the two rings is 1.30 (4)°. In the crystal structure, intermolecular O—H···N hydrogen bonds (Table 1) link the molecules in chains along the *b* axis.

### Experimental

A mixture of 1-(4-fluorophenyl)-2-(1*H*-imidazol-1-yl)ethanone (2.04 g, 10 mol), sodium borohydride (0.756 g, 20 mmol) and 30 ml dry ethanol was refluxed for 3 h. After solvent evaporation, the mixture was neutralized with dilute hydrochloric acid and then refluxed for 30 min. After the mixture was cooled, the solution was alkalinized with sodium hydroxide, the precipitate was collected, recrystallized with ethanol, and a yellow deposit was obtain (m.p. 410–412 K). Crystals suitable for a X-ray analysis were obtained by dissolving the crude product (1.0 g) in ethanol (30 ml) and then allowing the solution to evaporate slowly at room temperature for about 7 d.

### Refinement

The H atom of the hydroxy group was located in a difference Fourier map and was freely refined with *U*_iso_(H) = 1.5*U*_eq_(O). The other H atoms were positioned geometrically with C—H = 0.93 Å (aromatic), 0.97 Å (methylene), and 0.98 Å (methine) and constrained to ride on their parent atoms with *U*_iso_(H) = 1.2*U*_eq_(C). The absolute structure parameter is meaningless because the compound is a weak anomalous scatterer (Z < Si, MoKα). The Friedel-pair data were merged and the absolute structure parameter was removed from the CIF.

### Crystal data

**Table:**

C_11_H_11_FN_2_O	*F*(000) = 216
*M**_r_* = 206.22	*D*_x_ = 1.368 Mg m^−^^3^
Monoclinic, *P*2_1_	Melting point: 410 K
Hall symbol: P 2yb	Mo *K*α radiation, λ = 0.71073 Å
*a* = 7.1220 (14) Å	Cell parameters from 25 reflections
*b* = 5.4690 (11) Å	θ = 9–14°
*c* = 12.981 (3) Å	µ = 0.10 mm^−^^1^
β = 98.13 (3)°	*T* = 293 K
*V* = 500.53 (19) Å^3^	Block, colourless
*Z* = 2	0.30 × 0.20 × 0.10 mm

### Data collection

**Table:**

Enraf–Nonius CAD-4 diffractometer	876 reflections with *I* > 2σ(*I*)
Radiation source: fine-focus sealed tube	*R*_int_ = 0.029
graphite	θ_max_ = 25.4°, θ_min_ = 1.6°
ω/2θ scans	*h* = 0→8
Absorption correction: multi-scan (North *et al.*, 1968)	*k* = −6→6
*T*_min_ = 0.970, *T*_max_ = 0.990	*l* = −15→15
1992 measured reflections	3 standard reflections every 200 reflections
1024 independent reflections	intensity decay: 1%

### Refinement

**Table:**

Refinement on *F*^2^	Primary atom site location: structure-invariant direct methods
Least-squares matrix: full	Secondary atom site location: difference Fourier map
*R*[*F*^2^ > 2σ(*F*^2^)] = 0.041	Hydrogen site location: inferred from neighbouring sites
*wR*(*F*^2^) = 0.124	H atoms treated by a mixture of independent and constrained refinement
*S* = 1.00	*w* = 1/[σ^2^(*F*_o_^2^) + (0.090*P*)^2^] where *P* = (*F*_o_^2^ + 2*F*_c_^2^)/3
1024 reflections	(Δ/σ)_max_ < 0.001
139 parameters	Δρ_max_ = 0.13 e Å^−^^3^
1 restraint	Δρ_min_ = −0.18 e Å^−^^3^

### Special details

**Table:**

Geometry. All e.s.d.'s (except the e.s.d. in the dihedral angle between two l.s. planes) are estimated using the full covariance matrix. The cell e.s.d.'s are taken into account individually in the estimation of e.s.d.'s in distances, angles and torsion angles; correlations between e.s.d.'s in cell parameters are only used when they are defined by crystal symmetry. An approximate (isotropic) treatment of cell e.s.d.'s is used for estimating e.s.d.'s involving l.s. planes.
Refinement. Refinement of *F*^2^ against ALL reflections. The weighted *R*-factor *wR* and goodness of fit *S* are based on *F*^2^, conventional *R*-factors *R* are based on *F*, with *F* set to zero for negative *F*^2^. The threshold expression of *F*^2^ > σ(*F*^2^) is used only for calculating *R*-factors(gt) *etc*. and is not relevant to the choice of reflections for refinement. *R*-factors based on *F*^2^ are statistically about twice as large as those based on *F*, and *R*- factors based on ALL data will be even larger.

### Fractional atomic coordinates and isotropic or equivalent isotropic displacement parameters (Å^2^)

**Table:**

	*x*	*y*	*z*	*U*_iso_*/*U*_eq_	
F	0.9055 (3)	0.3909 (5)	0.52854 (19)	0.0796 (8)	
O	0.2786 (4)	−0.0461 (4)	0.19125 (18)	0.0557 (7)	
H0A	0.275 (6)	−0.068 (9)	0.125 (3)	0.078*	
N1	−0.0381 (3)	0.2845 (5)	0.16087 (19)	0.0445 (6)	
C1	0.5787 (5)	0.4661 (7)	0.2920 (3)	0.0524 (8)	
H1A	0.5509	0.5790	0.2384	0.063*	
C2	0.7277 (5)	0.5097 (7)	0.3702 (3)	0.0575 (9)	
H2A	0.8026	0.6486	0.3689	0.069*	
N2	−0.2416 (4)	0.3439 (6)	0.0183 (2)	0.0558 (8)	
C3	0.7627 (5)	0.3446 (7)	0.4495 (3)	0.0528 (9)	
C4	0.6583 (5)	0.1347 (7)	0.4532 (3)	0.0554 (9)	
H4A	0.6854	0.0246	0.5080	0.066*	
C5	0.5115 (5)	0.0912 (7)	0.3732 (2)	0.0479 (8)	
H5A	0.4397	−0.0507	0.3739	0.057*	
C6	0.4702 (4)	0.2556 (6)	0.2925 (2)	0.0412 (7)	
C7	0.3052 (4)	0.2069 (6)	0.2080 (2)	0.0426 (7)	
H7A	0.3311	0.2836	0.1432	0.051*	
C8	0.1259 (4)	0.3188 (7)	0.2395 (2)	0.0499 (8)	
H8A	0.1010	0.2448	0.3041	0.060*	
H8B	0.1460	0.4924	0.2518	0.060*	
C9	−0.1609 (5)	0.0911 (7)	0.1518 (3)	0.0538 (9)	
H9A	−0.1592	−0.0411	0.1970	0.065*	
C10	−0.2855 (5)	0.1288 (8)	0.0645 (3)	0.0538 (9)	
H10A	−0.3853	0.0254	0.0395	0.065*	
C11	−0.0933 (5)	0.4306 (7)	0.0793 (3)	0.0518 (8)	
H11A	−0.0336	0.5770	0.0672	0.062*	

### Atomic displacement parameters (Å^2^)

**Table:**

	*U*^11^	*U*^22^	*U*^33^	*U*^12^	*U*^13^	*U*^23^
F	0.0595 (12)	0.0851 (18)	0.0844 (15)	0.0039 (12)	−0.0237 (11)	−0.0152 (13)
O	0.0673 (15)	0.0462 (14)	0.0498 (13)	0.0048 (12)	−0.0049 (12)	−0.0048 (11)
N1	0.0383 (13)	0.0482 (15)	0.0465 (14)	0.0036 (13)	0.0049 (11)	0.0024 (12)
C1	0.0580 (19)	0.0450 (19)	0.0544 (18)	0.0012 (16)	0.0083 (16)	0.0045 (15)
C2	0.050 (2)	0.0474 (18)	0.074 (2)	−0.0045 (16)	0.0051 (17)	−0.0051 (19)
N2	0.0467 (15)	0.065 (2)	0.0539 (15)	0.0065 (14)	0.0007 (12)	0.0065 (15)
C3	0.0438 (17)	0.054 (2)	0.0572 (19)	0.0073 (16)	−0.0035 (14)	−0.0126 (17)
C4	0.063 (2)	0.056 (2)	0.0449 (18)	0.0105 (18)	−0.0012 (15)	0.0034 (16)
C5	0.0486 (17)	0.0496 (19)	0.0452 (16)	0.0010 (16)	0.0057 (14)	0.0024 (15)
C6	0.0384 (15)	0.0433 (18)	0.0426 (16)	0.0063 (13)	0.0084 (12)	−0.0042 (13)
C7	0.0459 (16)	0.0440 (17)	0.0374 (15)	0.0043 (13)	0.0041 (13)	0.0003 (13)
C8	0.0492 (18)	0.053 (2)	0.0474 (17)	0.0050 (17)	0.0047 (14)	−0.0054 (16)
C9	0.0556 (19)	0.053 (2)	0.0545 (19)	−0.0056 (16)	0.0128 (16)	0.0074 (16)
C10	0.0409 (16)	0.066 (2)	0.0550 (18)	−0.0048 (16)	0.0077 (15)	−0.0031 (18)
C11	0.0437 (16)	0.0496 (19)	0.062 (2)	0.0009 (16)	0.0054 (15)	0.0104 (16)

### Geometric parameters (Å, °)

**Table:**

F—C3	1.362 (4)	C4—C5	1.387 (5)
O—C7	1.409 (5)	C4—H4A	0.9300
O—H0A	0.86 (4)	C5—C6	1.380 (5)
N1—C11	1.340 (4)	C5—H5A	0.9300
N1—C9	1.367 (5)	C6—C7	1.514 (4)
N1—C8	1.450 (4)	C7—C8	1.523 (4)
C1—C2	1.382 (5)	C7—H7A	0.9800
C1—C6	1.387 (5)	C8—H8A	0.9700
C1—H1A	0.9300	C8—H8B	0.9700
C2—C3	1.365 (5)	C9—C10	1.353 (5)
C2—H2A	0.9300	C9—H9A	0.9300
N2—C11	1.316 (4)	C10—H10A	0.9300
N2—C10	1.376 (5)	C11—H11A	0.9300
C3—C4	1.372 (6)		
			
C7—O—H0A	106 (3)	C1—C6—C7	121.2 (3)
C11—N1—C9	106.3 (3)	O—C7—C6	111.0 (3)
C11—N1—C8	126.6 (3)	O—C7—C8	109.6 (3)
C9—N1—C8	127.0 (3)	C6—C7—C8	109.2 (2)
C2—C1—C6	120.6 (3)	O—C7—H7A	109.0
C2—C1—H1A	119.7	C6—C7—H7A	109.0
C6—C1—H1A	119.7	C8—C7—H7A	109.0
C3—C2—C1	118.6 (4)	N1—C8—C7	112.4 (3)
C3—C2—H2A	120.7	N1—C8—H8A	109.1
C1—C2—H2A	120.7	C7—C8—H8A	109.1
C11—N2—C10	105.0 (3)	N1—C8—H8B	109.1
C2—C3—F	118.8 (3)	C7—C8—H8B	109.1
C2—C3—C4	122.5 (3)	H8A—C8—H8B	107.9
F—C3—C4	118.6 (3)	C10—C9—N1	106.9 (3)
C3—C4—C5	118.2 (3)	C10—C9—H9A	126.6
C3—C4—H4A	120.9	N1—C9—H9A	126.6
C5—C4—H4A	120.9	C9—C10—N2	109.5 (3)
C6—C5—C4	120.9 (3)	C9—C10—H10A	125.3
C6—C5—H5A	119.5	N2—C10—H10A	125.3
C4—C5—H5A	119.5	N2—C11—N1	112.4 (3)
C5—C6—C1	119.1 (3)	N2—C11—H11A	123.8
C5—C6—C7	119.7 (3)	N1—C11—H11A	123.8
			
C6—C1—C2—C3	−1.7 (5)	C1—C6—C7—C8	−89.0 (3)
C1—C2—C3—F	−177.9 (3)	C11—N1—C8—C7	−87.2 (4)
C1—C2—C3—C4	1.5 (5)	C9—N1—C8—C7	89.0 (4)
C2—C3—C4—C5	−0.3 (5)	O—C7—C8—N1	−59.6 (4)
F—C3—C4—C5	179.0 (3)	C6—C7—C8—N1	178.6 (3)
C3—C4—C5—C6	−0.6 (5)	C11—N1—C9—C10	0.1 (3)
C4—C5—C6—C1	0.3 (5)	C8—N1—C9—C10	−176.7 (3)
C4—C5—C6—C7	−178.1 (3)	N1—C9—C10—N2	0.2 (4)
C2—C1—C6—C5	0.9 (5)	C11—N2—C10—C9	−0.5 (4)
C2—C1—C6—C7	179.3 (3)	C10—N2—C11—N1	0.6 (4)
C5—C6—C7—O	−31.6 (4)	C9—N1—C11—N2	−0.5 (4)
C1—C6—C7—O	150.0 (3)	C8—N1—C11—N2	176.3 (3)
C5—C6—C7—C8	89.4 (3)		

### Hydrogen-bond geometry (Å, °)

**Table:**

*D*—H···*A*	*D*—H	H···*A*	*D*···*A*	*D*—H···*A*
O—H0A···N2^i^	0.87 (4)	1.90 (4)	2.762 (4)	171 (5)

Symmetry codes: (i) −*x*, *y*−1/2, −*z*.
